# Asthma control cost-utility randomized trial evaluation (ACCURATE): the goals of asthma treatment

**DOI:** 10.1186/1471-2466-11-53

**Published:** 2011-11-24

**Authors:** Persijn J Honkoop, Rik JB Loymans, Evelien H Termeer, Jiska B Snoeck-Stroband, Moira J Bakker, Willem JJ Assendelft, Peter J Sterk, Gerben ter Riet, Tjard RJ Schermer, Jacob K Sont

**Affiliations:** 1 Dept of Medical Decision Making Leiden University Medical Center (LUMC) P.O. Box 9600 2300 RC Leiden, The Netherlands; 2 Dept of Public Health and Primary Care Leiden University Medical Center (LUMC) P.O. Box 9600 2300 RC Leiden, The Netherlands; 3 Dept of General Practice and Dept of Respiratory Medicine Academic Medical Center-University of Amsterdam (AMC) P.O. Box 22700 1100 DE Amsterdam, The Netherlands; 4 Dept of Primary and Community Care Radboud University Nijmegen Medical Centre (RUNMC) P.O. Box 9101 6500 HB Nijmegen, The Netherlands; 5 Dept of Respiratory Medicine Academic Medical Center-University of Amsterdam (AMC) P.O. Box 22700 1100 DE Amsterdam, The Netherlands

## Abstract

**Background:**

Despite the availability of effective therapies, asthma remains a source of significant morbidity and use of health care resources. The central research question of the ACCURATE trial is whether maximal doses of (combination) therapy should be used for long periods in an attempt to achieve complete control of all features of asthma. An additional question is whether patients and society value the potential incremental benefit, if any, sufficiently to concur with such a treatment approach. We assessed patient preferences and cost-effectiveness of three treatment strategies aimed at achieving different levels of clinical control:

1. sufficiently controlled asthma

2. strictly controlled asthma

3. strictly controlled asthma based on exhaled nitric oxide as an additional disease marker

**Design:**

720 Patients with mild to moderate persistent asthma from general practices with a practice nurse, age 18-50 yr, daily treatment with inhaled corticosteroids (more then 3 months usage of inhaled corticosteroids in the previous year), will be identified via patient registries of general practices in the Leiden, Nijmegen, and Amsterdam areas in The Netherlands. The design is a 12-month cluster-randomised parallel trial with 40 general practices in each of the three arms. The patients will visit the general practice at baseline, 3, 6, 9, and 12 months. At each planned and unplanned visit to the general practice treatment will be adjusted with support of an internet-based asthma monitoring system supervised by a central coordinating specialist nurse. Patient preferences and utilities will be assessed by questionnaire and interview. Data on asthma control, treatment step, adherence to treatment, utilities and costs will be obtained every 3 months and at each unplanned visit. Differences in societal costs (medication, other (health) care and productivity) will be compared to differences in the number of limited activity days and in quality adjusted life years (Dutch EQ5D, SF6D, e-TTO, VAS). This is the first study to assess patient preferences and cost-effectiveness of asthma treatment strategies driven by different target levels of asthma control.

**Trial registration:**

Netherlands Trial Register (NTR): NTR1756

## Background

Despite the availability of effective therapies, asthma remains a source of significant morbidity and use of health care resources [1,2]. The societal costs of asthma are considerable. Asthma negatively affects work productivity as well as labour force participation. Furthermore, a survey showed that in the Netherlands 30% of asthmatics needed urgent care in the past year, which was on average 8% more than in other European countries [3]. Under a system designed for acute rather than chronic care, patients are not adequately taught to care for their own illness. Sixty-two percent of patients visit their pulmonary specialists or general practitioners only if they have an acute health problem. Only 15% of Dutch asthmatics had a doctor-written action plan for their asthma [3]. In addition, there is a major discrepancy between patients' perceived control of asthma and symptom severity [4]. National and international guidelines define the goal of treatment as to achieve and maintain clinical asthma control [5,6]. Daily treatment with inhaled corticosteroids is recommended on a long-term basis as first-line therapy to keep asthma under clinical control in patients with persistent asthma. Short-term bronchodilators are used on an as-needed basis to reverse bronchoconstriction and relieve symptoms. The 2006 updated international guidelines [6] introduced a management approach based on asthma control. According to the Global Initiative for Asthma (GINA) guidelines the levels of asthma control are defined as follows:

1) Partly controlled asthma is defined as the presence of any of the following: daytime symptoms ≥ twice per week, limitations of activities, nocturnal symptoms, need for reliever treatment, reduced lung function and exacerbations (further referred as sufficiently controlled).

2) Controlled asthma is defined as daytime symptoms that are present ≤ twice per week or the absence of limitations of activities, nocturnal symptoms, need for reliever treatment, reduced lung function and exacerbations (further referred as strictly controlled).

3) Uncontrolled asthma is defined as ≥ 3 features of partly controlled or the presence of an exacerbation.

The level of asthma control can be assessed using composite measures such as the validated Asthma Control Questionnaire (ACQ) [7]. Each patient should be assessed to establish the current treatment regimen, adherence to the current regimen, and the level of asthma control. If asthma is uncontrolled on the current treatment regimen, treatment should be stepped up until control is achieved. If asthma is partly controlled, the guidelines recommend that a step-up in treatment should be considered.

Strictly controlled asthma can be achieved in the majority of patients with uncontrolled asthma by a treatment strategy with (high dose) inhaled corticosteroids alone or with combination therapy of an inhaled corticosteroid and a long-acting bronchodilator [8]. Symptoms and lung function will improve and the number of awakenings and severe exacerbation rate will reduce [9]. However, this is in marked contrast with the levels of control observed in community studies, where patients tend to be partly controlled [4]. Current guidelines show some ambiguity whether treatment target should be controlled or partly controlled [6]. Another question is not only whether maximal doses of (combination) therapy should be used for long periods in an attempt to achieve complete control of all features of asthma, but also whether patients would value the potential incremental benefit sufficiently to concur with such a treatment approach [10]. In addition, there is only limited data available on the cost-effectiveness of treatment strategies aimed at different levels of asthma control [11,12].

Recently, the fraction of exhaled nitric oxide (FeNO) has been introduced as a non-invasive marker of airway inflammation in asthma. The role of FeNO in titrating anti-inflammatory treatment to the most effective dose of inhaled corticosteroids in asthma is still controversial [13]. Addition of FeNO as an indicator of control of asthma has led to higher [14] as well as lower [15] doses of inhaled corticosteroids without a difference in symptomatic asthma control. Adjustments to medication dose based on FeNO measurements seem to reduce the number of exacerbations, but recent studies had insufficient power to reach statistical significance when adjusting for multiple exacerbations within patients [16]. Therefore, it is not yet determined whether FeNO measurements may indicate whether a step-up in treatment is effective or a step-down can be achieved without loss of asthma control and thereby contribute to the efficiency of asthma care.

Therefore, we aim to investigate whether a treatment strategy aimed at strict asthma control is more (cost-)effective as compared to a treatment strategy aimed at achieving sufficiently controlled asthma. In addition we postulate that a treatment strategy aimed at strict asthma control is more (cost-)effective when the treatment step is additionally guided by measurements of exhaled nitric oxide (FeNO) as compared to a treatment strategy aimed at achieving strictly controlled asthma or sufficiently controlled asthma without the addition of FeNO.

### Preliminary results

#### Monitoring control

An internet application will be used to assist the physician/nurse practitioner/physician assistant in adjusting the treatment step according to the 3 treatment algorithms. In the Self-Management of Asthma Supported by Hospitals, Information and communication technology, Nurses and General practitioners (SMASHING) -project we have already used an internet application for monitoring Forced Expiratory Volume in 1 second (FEV_1_) and asthma control questionnaire (ACQ) [17]. Furthermore, in this project we have set-up electronic versions http://www.netquestionnaires.nl of the majority of questionnaires. In the OPPAS-project (UMCN) we have already explored the distribution of levels of asthma control in general practice patients with asthma [18].

### Design

**Figure 1:**
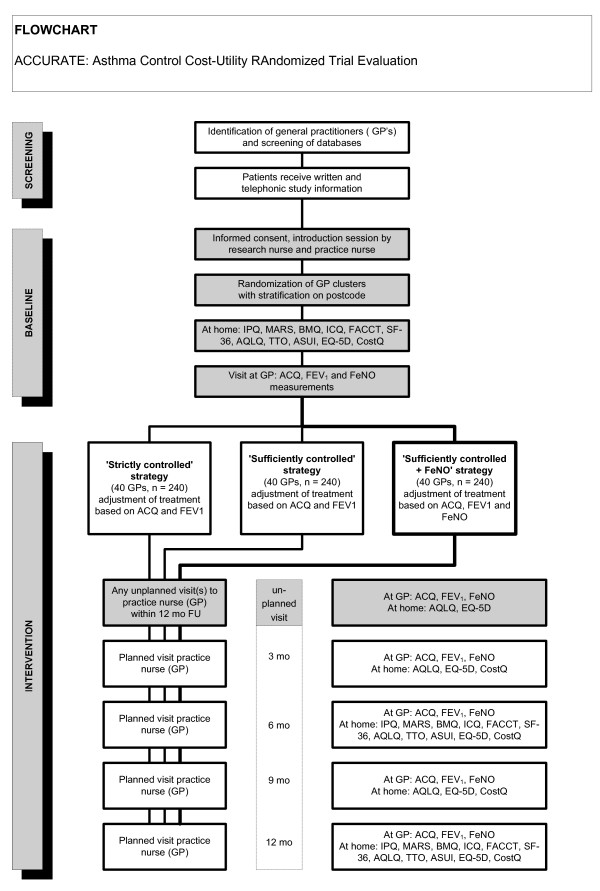
**Flowchart of the ACCURATE trial**.

The study is a cluster-randomised parallel trial with 3 arms and 12 months follow-up (Figure 1). In order to avoid recruitment bias the identification of potential patients from the general practice information system will be performed before the allocation of a general practice cluster to a treatment strategy [19,20]. The 3 treatment strategies are defined as:

1 SUFF-strategy: achieving sufficiently controlled asthma based on conventional asthma control measures

2 STRICT-strategy: achieving strictly controlled asthma based on conventional asthma control measures

3 FeNO-strategy: achieving strictly controlled asthma based on conventional asthma control measures and an indirect marker of airways inflammation (FeNO).

General practices will be randomly assigned to the 3 groups using a computer generated permuted block scheme, ensuring concealment of allocation. Treatment assignment will be stratified according to characteristics of general practices (solo/duo/etc practice, rural/urban). The patients will visit the general practice for an introduction visit and control visits at baseline, 3, 6, 9, and 12 months. In case of asthma exacerbations patients pay an additional visit to the general practice or chest physician.

#### Intervention

The level of asthma control will be based on a 3-monthly assessment of asthma symptoms, number and severity of exacerbations, FEV_1_, with or without the level of FeNO. Asthma symptoms will be assessed with the ACQ, which is closely associated with the level of asthma control from the GINA guidelines (Table 1). Step-ups in medication will be adjusted (Table 2), using specific algorithms for the 3 treatment strategies (Table 3). The step-up in medication in the FeNO-strategy will be additionally guided by the level and change in FeNO according to recent recommendations and the latest available evidence [21]. This allows adjustment of the dosage of inhaled corticosteroids based on information of airways inflammation whilst the dosage of additional reliever medication is based on asthma control measures [21]. At each planned and unplanned visit during the 12 months follow-up maintenance, therapy will be adjusted according to the relevant algorithm, using the internet-based asthma monitoring system by either the nurse practitioner or general practitioner [17]. This allows the supervision of this process by a central coordinating nurse specialist.

**Table 1:** Levels of Asthma Control

Characteristic	Strictly Controlled (All of the following)	Sufficiently Controlled (Any measure present in any week)	Uncontrolled
Daytime symptoms	None (twice or less/week)	More than twice/week	Three or more features of sufficiently controlled asthma present in any week
Limitations of activities	None	Any	
Nocturnal symptoms/awakening	None	Any	
Need for reliever/rescue treatment	None (twice or less/week)	More than twice/week	
Lung function (FEV_1_)	Normal	< 80% predicted	
Exacerbations*	None	1^st ^moderate exacerbation	≥ 2 moderate exacerbation^† ^or severe exacerbation

*modified from the GINA guidelines; the presence of an exacerbation influences the level of asthma control at baseline or at an exacerbation. If one or more exacerbations have led to an adjustment in treatment, this category starts at 0 again. At baseline: treatment levels only will be adjusted when exacerbations were present ≤ 3 months prior to the study: † during the same treatment regime.

**Table 2:** Management approach based on control (GINA guidelines)

STEP 1	STEP 2	STEP 3	STEP 4	STEP 5
**Asthma education**				
**Environmental control**				

**As needed rapid-acting ß_2_-agonist**	**As needed rapid-acting ß_2_-agonist**			
	**Select one**	**Select one**	**Add one or more**	**Add one or both**

	Low-dose ICS*	Low-dose ICS plus long-acting ß_2_-agonist	Medium- or high dose ICS plus long-acting ß_2_-agonist	Oral corticosteroid (lowest dose)
	Leukotriene modifier	Medium- or high dose ICS	Leukotriene modifier	Anti-IgE treatment
		Low-dose ICS plus Leukotriene modifier^†^		

*ICS = inhaled corticosteroids

**Table 3:** Treatment strategy algorithms

	*Levels of asthma control*		
Strategy	*Strictly controlled*	*Sufficiently controlled*	*Uncontrolled*
**STRICT-strategy**	- 3 mo: no change	step-up: treatment choice	step-up: treatment choice
	- > 3 mo: step-down		

**SUFF-strategy**	step-down	no change	step-up: treatment choice

**FeNO strategy**			
*- Low FeNO level*	step-down	- 3 mo: no change/change within current step to LABA	step-up: LABA
		- > 3 mo: step-down ICS	
*- Intermediate FeNO level*	no change	step-up: treatment choice	step-up: treatment choice
*- High FeNO level*	step-up/change within current step to ICS	step-up: 1 × ICS	step-up: 2 × ICS*

STRICT-strategy = strictly controlled strategy; SUFF-strategy = sufficiently controlled strategy. A raised FeNO level is indicative of eosinophilic airway inflammation; ICS = inhaled corticosteroids; LABA = long-acting ß_2_-agonist. *until a maximum high dose of ICS is reached

#### Patients

720 Patients with mild to moderate persistent asthma (prevalent cases) will be recruited from general practices via patient registries in three regions in The Netherlands:

- Leiden University Medical Center (LUMC) general practice network LEON (240 patients, 40 general practices)

- Radboud University Nijmegen Medical Centre general practice network (240 patients, 40 general practices)

- Amsterdam Medical Center (AMC) general practice network (240 patients, 40 general practices)

Only general practices with a practice nurse (or nurse practitioner/or physician assistant, further referred to as 'practice nurse') will participate (70% of general practices in the Netherlands currently have a employed such a professional [22]). Based on previous research experience in patients with asthma in general practice (SMASHING-project CME number P05.136), we estimate a response rate of 40% with an inclusion of 6 patients per 'standard' practice (i.e. practice with 2,300 patients) and 40 'standard' general practices per treatment strategy.

##### Inclusion criteria

(all of the following criteria)

- age 18-50 yr

- doctor's diagnosis of asthma

- patients who need inhaled corticosteroids as controller medication (step 2-4 GINA guidelines)

- inhaled corticosteroids ≥ 3 months in the previous year

- written informed consent

- no exacerbation of asthma within 1 month before entry

##### Exclusion criteria

- daily or alternate day oral corticosteroid therapy for at least 1 month before entering into the study

- inability to understand written and oral Dutch instructions

- active diseases likely to interfere with the purpose of the study, such as end-stage disease or inability to visit the general practitioner

#### Methods of measurements

At baseline, patient characteristics will be assessed including questions on atopy, smoking and symptom free days. In line with the Dutch national guidelines for general practitioners [3], patients are invited to visit their general practice every 3 months in order to titrate medication to the lowest level that is needed to achieve or maintain control. 3-Monthly care by the nurse practitioner will be organized similar to the advise in the national guidelines for general practitioners [5], including questions on asthma control, medication, adverse events and measurement of lung function. At all planned and unplanned visits questionnaires will be performed at home (Table 4). In addition, the ACQ will be performed at home monthly as an outcome measure. Peripheral blood will be obtained at baseline. Both paper and electronic versions will be used to collect the data, depending on the preference of a patient. Electronic versions in the ACCURATE project will be similar to those from the SMASHING project http://www.netquestionnaires.nl. A coordinating nurse specialist will supervise the nurse practitioners.

**Table 4:** Instrument Table

	Assessment of level of asthma control:			Outcomes									
	driving treatment step												
				Patient preferences			Quality of life			Patient utilities			Costs
	airway inflammation	lung function	asthma control	beliefs about medicines	adherence	adherence	patient statisfaction	generic	asthma related	patient perspective	asthma related	societal perspective	health care + other costs
	FeNO	FEV_1_	ACQ	IPQ	MARS	BMQ	FACCT	SF-36	AQLQ	TTO	ASUI	EQ-5D	CostQ
**Baseline**	X	X	X	X	X	X	X	X	X	X	X	X	X
**Unplanned visit**	F	X	X						X		X	X	
**3 months**	F	X	X						X		X	X	X
**6 months**	F	X	X	X	X	X	X	X	X	X	X	X	X
**9 months**	F	X	X						X		X	X	X
**12 months**	X	X	X	X	X	X	X	X	X	X	X	X	X

X in all treatment strategies, F only in FeNO strategy

##### Assessment of level of asthma control

At each planned and unplanned visit to the general practice a nurse practitioner will assess the level of asthma control with:

1. asthma control questionnaire (ACQ-score) [7]

2. lung function level (FEV_1_)

3. FeNO (only in the FeNO-strategy)

4. presence of exacerbations, now or in previous weeks

##### Asthma control questions

Asthma control will be assessed every 3 months with the Asthma Control Questionnaire (ACQ), which consists of 6 items with a 7-point scale (0 = totally controlled, 6 = severely uncontrolled) [7]. In addition, the ACQ will be completed monthly at home. The ACQ contains questions on respiratory symptoms over the previous week. The patients will be asked whether these symptoms were representative for the last 4 weeks. If not, the ACQ will be assessed from the most representative of the last 4 weeks. The optimal cut-point for "strictly controlled" asthma is defined as a mean ACQ score ≤ 0.75 and a score of ≥1.50 confirms "uncontrolled" asthma [23]. We regard control to be sufficient if 0.75 < mean ACQ < 1.50.

##### Lung function measurements

Spirometry will be performed in the general practices according to national [5] and international guidelines [24]. For the baseline visit patients will be instructed to refrain from bronchodilator use for a specified number of hours before the scheduled spirometry test. Reversibility of airways obstruction will be measured 20 min after administering 4 single puffs of 100 μg salbutamol per metered dose-inhaler connected to a spacer (Volumatic^®^). The response will be expressed as ml and percentage change in predicted value of FEV_1_.

##### Exhaled nitric oxide

Fraction of exhaled Nitric Oxide (FeNO) will be measured in the general practices according to international guidelines [25] with the NIOX-MINO (Aerocrine, Solna, Sweden) [26]. At baseline and at the last visit all patients will perform FeNO measurement, whereas at 3, 6, and 9 months FeNO only will be assessed in the FeNO Group. FeNO will be measured before spirometric manoeuvres, at an exhaled rate of 50 ml/sec maintained for 10 seconds. Patients are not allowed to smoke at least one hour before the measurements. Results are expressed as the NO concentration in ppb (equivalent to nanolitres/litre) based on the first approved measurement. FeNO levels will be categorized into low when FeNO ≤ 25 ppb (absence of inflammation), intermediate 25 ppb < FeNO < 50 ppb and high FeNO ≥ 50 ppb (presence of airway inflammation) [13]. Results will be adjusted for smoking (yes/no), gender and height [27].

##### Exacerbations

Patients will be instructed to pay an additional visit to their general practice if they experience worsening of asthma symptoms. In line with the national [5] and GINA guidelines [6] exacerbations of asthma are defined as acute or subacute episodes of progressively worsening shortness of breath, cough, wheezing, and chest tightness, or some combination of these symptoms [28] and will be treated by the general practitioner [5]. FeNO will be performed only in the FeNO strategy. Additional questionnaires and lung function will be performed at home (Table 4).

After an exacerbation is resolved the patient visits the nurse practitioner who will assess the current level of asthma control. GINA is not clear about the incorporation of exacerbations in the assessment of asthma control (Table 4.3-1 from the GINA guidelines; 'personal communication'), but in the same GINA document exacerbations are also referred to as evidence of poor asthma control. Therefore, in the present study exacerbations are handled as follows. Questions will be asked on new respiratory symptoms, medication change and hospitalisation [28]. The exacerbation will be classified according to severity as based on the presence of respiratory symptoms, prescribed medication and/or hospitalisation. A moderate exacerbation is defined as a (sub)acute deterioration in symptoms and/or lung function with increased rescue bronchodilator use (or ICS) which lasts 2 days or more, not severe enough warrant oral steroids (for 3 days or more) or hospitalisation. A severe exacerbation is defined as (sub)acute deterioration in asthma resulting in the need for oral steroids for 3 days or more or hospitalisation (as judged by the physician) [29]. Subsequently, the level of control will be assessed as based on Table 1 and maintenance therapy will be assigned according to the treatment algorithm after exacerbation treatment is finished.

##### Assessment of cost-utilities and patient preferences Costs

- cost questionnaires: health care consumption; absenteeism and productivity loss (CostQ) [30]

##### Patient preferences

- the Foundation for Accountability (FACCT) [31]

- the Brief Illness Perception Questionnaire (Brief IPQ) [32]

- the Beliefs about Medicines Questionnaire (BMQ) [33]

- Medication Adherence Report Scale (MARS) [34,35]

##### Quality of life, patient utilities

- quality of life: Asthma Quality of Life Questionnaire (AQLQ) [36] and Short-Form 36 (SF-36) [37]

- patient utilities: the Asthma Symptom Utility Index (ASUI) [38]. Patient utilities will additionally be assessed by the time-trade-off method by telephonic interview and a web-page (e-TTO) at each planned and unplanned period (exacerbation) [39]

- indirect utilities from the general public will be obtained using the SF-6D [37,34] and EQ-5D [40,41]. This allows the calculation of quality adjusted life years (QALYs).

- number of limited activity days by questionnaire

#### Analysis

The analysis will be carried out on an intention to treat basis. The data set will consist of all included patients from randomised practices.

##### Sample-size calculation

**Figure 2:**
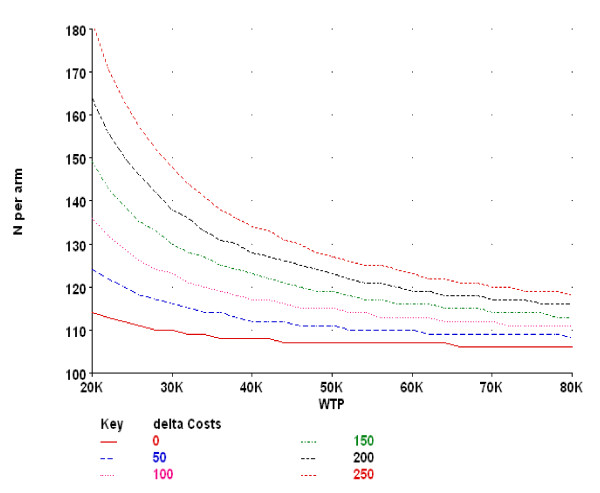
**Power curve of the required sample-size per treatment arm**. The curve is represented as a function of willingness-to-pay (WTP) for a range of increases in costs (delta Costs) when a treatment strategy is not only more effective but also more costly. The presented +sample-size is unadjusted for intra-cluster correlation. A minimally important change in patient utility (EQ-5D) has been defined as 0.074 point. With 150 patients per treatment strategy we are able to detect at least a change of 0.06 points by net health benefit analysis between the arms with a SD = 0.175 EQ-5D points (baseline data SMASHING-project: SD = 0.17) and a SD of €1000 for costs (SD = €816, usual care strategy) and an increase in costs of €250 (delta Costs) when a treatment strategy is not only more effective but also more costly, for a willingness-to-pay (WTP) of €30K (alpha = 0.05, one-sided, beta = 0.20, one-sided, rho costs-effects = 0). With 40 clusters (general practices) per arm and assuming an intra-cluster correlation of 0.01, 0.07 and 0.11 the number of patients per cluster is 4, 5, and 6, and the total number of patients is 480, 600 and 720, respectively.

A minimally important change in patient utility (EQ-5D) has been defined as 0.074 point [42]. With 150 patients per treatment strategy we are able to detect at least a change of 0.06 points by net health benefit analysis [43] between the arms with a SD = 0.175 EQ-5D points (baseline data SMASHING-project; trial registry number NTR826: SD = 0.17) and a SD of €1000 for costs (SD = €816, usual care strategy [44]) and an increase in costs of €250 when a treatment strategy is not only more effective but also more costly, for a willingness-to-pay (WTP) of €30K (alpha = 0.05, one sided [43], beta = 0.20, one sided, rho costs-effects = 0) (Figure 2). With 40 clusters (general practices) per arm and assuming an intra-cluster correlation of 0.01, 0.07 and 0.11 the number of patients per cluster is 4, 5, and 6, and the total number of patients is 480, 600 and 720, respectively [45].

##### Data-analysis and presentation/synthesis

At baseline, data from all planned and unplanned contact will be collected according to the scheme in Table 4. The instruments include variables of:

- *patient characteristics*: age, sex, socioeconomic status, smoking status and smoking history

- *medical outcomes*: FEV_1_, FeNO, ACQ, current treatment step, asthma medication

- *patient preferences*: FACCT, IPQ, BMQ, MARS

- *quality of life*: AQLQ, SF-36

- *patient utilities*: ASUI, SF-6D and EQ-5D, QALYs, e-TTO, number of limited activity days

- *costs*: health care consumption; CostQ

#### Economic evaluation

##### General considerations

The economic evaluation will compare differences in societal effects and costs to differences in the number of limited activity days (cost-effectiveness analysis, CEA) and quality adjusted life years (cost-utility analysis, CUA). The analysis will have a 12-months time horizon, without discounting. Group averages will be statistically compared using two-sided bootstrapping and net-benefit analysis will be used to relate costs to patient outcome. Sensitivity analyses will be performed on the perspective (societal versus health care) and the applied utility measure (Dutch EQ5D, SF6D, e-TTO, AQLQ-5D).

##### Cost-effectiveness

The primary end-point is the evaluation of the cost-effectiveness of treatment strategies by incremental net-benefit analysis [43]. Net health benefit addresses cost-effectiveness ratios by assuming values for the willingness-to-pay per unit of effectiveness.

##### Cost analysis

The cost analysis will include both medical (medication, visits, and hospitalizations) and non-medical costs (productivity losses, informal care). Purchased medication will be assessed from electronic patient records (with written patient permission), complemented with the patient's report on medication purchased elsewhere [46]. Other costs will be estimated using quarterly cost questionnaires (CostQ) [30]. Costs will be valued according to standard prices charges [47] including time and travel costs.

##### Analysis of effectiveness

The differences in levels and changes in utilities based on EQ-5D, SF-6D, VAS, e-TTO and the number of limited activity days will be compared between the treatment strategies using a random-effects analysis accounting for within-patient repeated measurements and clustering on general practice.

##### Patient outcome analysis

Utilities will be assessed every three months. In the base case analysis, quality-adjusted life years (QALYs) will be estimated using societal utilities obtained using the Dutch EQ5D tariff [48]. As sensitivity analyses, QALYs will be estimated using the SF-6D and individual utilities obtained using the e-TTO and visual analogue scale (transformed using a power transformation).

#### Ethical approval

Ethical approval was obtained from the Medical Ethics Committee of the Leiden University Medical Center (ABR no: 24488).

## Discussion

The aim of the ACCURATE trial is to compare the cost-effectiveness and patient preferences of three asthma treatment strategies: 1) sufficiently controlled strategy, aiming to achieve sufficiently controlled asthma based on conventional asthma control measures (ACQ and lung function); 2) strictly controlled strategy, aiming to achieve controlled asthma also based on asthma conventional control measures; and 3) FeNO-strategy, aimed at achieving strictly controlled asthma based on conventional asthma control measures plus an indirect marker of airways inflammation. For this purpose we will implement an internet-based programme, to be used by care providers in general practices.

To our knowledge, this is the first study to assess patient preferences and cost-effectiveness of asthma treatment strategies aimed at different levels of control on asthma. Notably, the current study is fully investigator driven, granted by governmental funding rather than pharmaceutical funding. Current guidelines advise clinicians to ensure that asthma is strictly controlled, i.e. patients should not experience any symptoms. However, in daily practice, a considerable proportion of asthma patients continuously experience symptoms without consulting their physician [49]. This raises the question of patient's preferences with regard to treatment aims. It is not yet known whether patients are willing to conform to the stringent treatment aim of strictly controlled asthma, especially if it results in high doses of asthma medication and an increased likelihood of concurrent side effects. These uncertainties hamper implementation of current guidelines and therefore a great diversity in treatment exists. Furthermore, discordance in patient's and medical treatment goals might result in unnecessary asthma symptoms and health care use.

A recent meta-analysis showed that FeNO guided treatment of asthma does not reduce the number of exacerbations; however it did reduce the daily dose of inhaled corticosteroids [50]. Our study may extend these findings by providing further understanding of the cost-effectiveness and patient preferences of FeNO guided treatment of asthma.

We hypothesize that:

1) a treatment strategy aimed achieving at strictly controlled asthma is more (cost-)effective as compared to a treatment strategy aimed at sufficiently controlled asthma;

2) a treatment strategy aimed at achieving strictly controlled asthma is (cost-)effective when the treatment step is additionally guided by measurements of exhaled nitric oxide (NO) as compared to a treatment strategy aimed at strictly controlled asthma or sufficiently controlled asthma.

During the conduction of the trial the definition of asthma exacerbations has been changed. In our analysis we will use the definitions as proposed by the ATS/ERS task force [51]. By incorporating internet-based graphic feedback on a patients' asthma control status and internet-based decision support based on current guidelines, we will enhance the feasibility and standardization of the treatment advice. The results of this study will provide insight into the potential discordance between patient's and medical treatment goals and the effect on health care costs from the societal perspective. The internet-based decision support methodology and results of our study may facilitate cost-effective implementation of future tailored treatment strategies for patients with mild to moderate asthma in primary care.

## Competing interests

The institute of PS has received a public-private EU grant from the Innovative Medicines Initiative in the field of asthma (U-BIOPRED) with Amsterdam as coordinating centre. JS received unrestricted research grants from GlaxoSmithKline (NL) and Astra-Zeneca (NL). All other authors declare that they have no competing interests.

## Authors' contributions

JKS, JBS, GtR, WA, PJS, TS, MB, PJH and EHT were involved in the design of the study; PJH, MB, EHT and RJL collected the data; JBS, PJH and RJL drafted this manuscript, which was revised by JKS, GtR, PS, EHT, WA and TS. All authors gave their final approval for publication.

## Pre-publication history

The pre-publication history for this paper can be accessed here:

http://www.biomedcentral.com/1471-2466/11/53/prepub
